# Chamber-specific long-term prognosis of intracardiac thrombus: a 21-year propensity-matched cohort study

**DOI:** 10.3389/fcvm.2026.1889091

**Published:** 2026-10-13

**Authors:** Victor Chien-Chia Wu, Chih-Shan Liu, Chun-Li Wang, Ya-Chi Huang, Hui-Tzu Tu, Hsin-Jui Liao, Yu-Tung Huang, Shao-Wei Chen, Chang-Fu Kuo, Shang-Hung Chang

**Affiliations:** 1 Division of Cardiology, Chang Gung Memorial Hospital, Linkou Medical Center, Taoyuan City, Taiwan; 2 College of Medicine, Chang Gung University, Taoyuan City, Taiwan; 3 Center for Big Data Analytics and Statistics, Chang Gung Memorial Hospital, Linkou Medical Center, Taoyuan City, Taiwan; 4 Information System Division, Chang Gung Medical Technology Co., Ltd., Taipei, Taiwan; 5 Department of Cardiothoracic and Vascular Surgery, Chang Gung Memorial Hospital, Linkou Medical Center, Taoyuan City, Taiwan; 6 Division of Rheumatology, Allergy and Immunology, Department of Internal Medicine, Chang Gung Memorial Hospital, Linkou Medical Center, Taoyuan City, Taiwan; 7 Division of Rheumatology, Orthopaedics and Dermatology, School of Medicine, University of Nottingham, Nottingham, United Kingdom; 8 Graduate Institute of Nursing, Chang Gung University of Science and Technology, Taoyuan City, Taiwan

**Keywords:** cardiac chamber, cardiovascular outcome, echocardiography, embolic risk, intracardiac thrombus

## Abstract

**Background:**

The prognostic impact of intracardiac thrombus varies by cardiac chamber, yet large-scale comparative long-term outcome data across all four chambers remain limited.

**Methods:**

In this retrospective propensity-matched cohort study using the Chang Gung Research Database (2001–2022), we identified 1,541 patients in the final propensity-matched analytic cohorts with echocardiographically confirmed single-chamber intracardiac thrombus [left atrial (LA) *n* = 619, left ventricular (LV) *n* = 774, right atrial (RA) *n* = 125, right ventricular (RV) *n* = 23]. Patients were 1:4 matched with controls without thrombus for demographics, comorbidities, and medications. Primary outcomes included ischemic stroke/systemic embolism, pulmonary embolism, myocardial infarction, heart failure, cardiac death, and major adverse cardiovascular events (MACE; composite of the above). Follow-up continued until the first event, death, or December 31, 2022.

**Findings:**

Compared with matched controls, LA thrombus was associated with increased risks of ischemic stroke/systemic embolism (hazard ratio, HR = 1.85), heart failure (HR = 1.30), cardiac death (HR = 2.02), and MACE (HR = 1.61). LV thrombus was associated with increased risks of ischemic stroke/systemic embolism (HR = 1.38), myocardial infarction (HR = 1.58), heart failure (HR = 1.40), cardiac death (HR = 1.32), and MACE (HR = 1.36). RA thrombus was associated with pulmonary embolism (HR = 4.63), heart failure (HR = 1.70), and MACE (HR = 1.63), whereas cardiac death was not significantly increased (HR = 1.96). RV thrombus was rare and showed no significant associations with any outcome. Left atrial appendage thrombus did not confer additional risk compared with non-appendage LA thrombus.

**Conclusions:**

The prognostic impact of intracardiac thrombus is strongly location-dependent. LV and LA thrombi carried the broadest cardiovascular risks, RA thrombus was disproportionately linked to pulmonary embolism and mortality, and RV thrombus had limited long-term impact. These findings support chamber-specific risk stratification and management strategies for patients with echocardiography-detected intracardiac thrombus.

## Introduction

Cardiac chamber thrombus is a clinically significant entity arising from diverse cardiovascular and systemic conditions. Depending on the underlying pathophysiology, thrombi can form in any of the four chambers and may lead to embolic complications involving the cerebral, coronary, or systemic circulation. Left-sided thromboembolism alone is responsible for an estimated 15%–20% of ischemic strokes (IS) in the United States, underscoring the importance of early recognition and management (1). Echocardiography plays a central role in the detection and anatomic characterization of intracardiac thrombi (2).

The mechanisms of thrombus formation vary by chamber. Left atrial (LA) thrombus typically results from blood stasis in settings such as mitral stenosis or atrial fibrillation, while left ventricular (LV) thrombus often develops in the context of reduced contractility or regional wall motion abnormalities in heart failure (HF) or myocardial infarction (MI) (3–7). Thrombi in the right atrium (RA) or right ventricle (RV) are frequently associated with hypercoagulable states, including hematologic, autoimmune, or malignant diseases, and may present as clot-in-transit with concomitant deep vein thrombosis or pulmonary embolism (PE) (8–11). Thrombus formation may also occur on prosthetic valves or cardiac devices following implantation (12, 13).

Although these etiologies are recognized, the prognostic implications of thrombus according to its specific cardiac chamber remain inadequately defined. In particular, comparative data on cardiovascular outcomes such as stroke, systemic embolism (SE), myocardial infarction, heart failure, cardiac death, and composite major adverse cardiovascular events (MACE) across chambers are limited. To address this knowledge gap, we analyzed a large multi-institutional echocardiography database to evaluate outcomes associated with thrombus in each of the four cardiac chambers.

## Methods

### Data source

In this retrospective cohort study, patient data were obtained from the largest healthcare provider in Taiwan, Chang Gung Memorial Hospital System, comprising three major teaching hospitals and four tertiary-care medical centers (14–16). The healthcare provider has more than 10,000 beds, admits more than 280,000 patients, and serves approximately one-tenth of the Taiwan's population each year. To protect patient privacy, hospital identification numbers were encrypted and de-identified. Therefore, informed consent was waived for this study. Diagnostic and laboratory data were linked and continuously monitored using consistent data encryption. The institutional review board of Chang Gung Memorial Hospital approved the study protocol (IRB No. 201901586B0).

### Study patients

By searching electronic medical records from the Chang Gung Memorial Hospital System (Chang Gung Research Database) between 1 January 2001 and 31 January 2022, we identified patients with cardiac thrombus formation documented in transthoracic echocardiography (TTE) and transesophageal echocardiography (TEE) reports. We included patients who had received at least 30 days of oral, subcutaneous, or intravenous anticoagulation as confirmation. We excluded patients if thrombus location was not specified, if thrombus was situated inside pericardium, pulmonary vein, aorta, vena cava, or pulmonary artery, or if thrombi involved more than one cardiac chamber (i.e., both RA and RV chambers). Eligible patients were categorized as having LA, LV, RA, or RV thrombus according to the chamber involved.

### Determination of location of cardiac thrombus

To determine the anatomic location of cardiac thrombus, echocardiographic reports were reviewed and adjudicated by a physician with more than 10 years of experience in performing and interpreting cardiac ultrasound according to prespecified criteria. Thrombi situated in the LA or attached to the mitral valve were classified as LA thrombi; thrombi situated in the LV or attached to the aortic valve were classified as LV thrombi; thrombi situated in the RA or attached to the tricuspid valve were classified as RA thrombi; and thrombi situated in the RV or attached to the pulmonic valve were classified as RV thrombi. Thrombi situated in the pericardium, vena cava, pulmonary vein, pulmonary artery, or aorta, as well as thrombi involving more than one cardiac chamber, were excluded.

### Study outcomes and follow-up

Primary outcomes were IS/SE, PE, MI, HF (new onset or acute decompensation), cardiac death, and MACE (composite of IS/SE, PE, MI, HF, and cardiac death), defined according to the principal diagnosis of an admission or emergency visit. Patients were followed independently for each outcome until the first occurrence of that outcome, death, or the end of follow-up. Death was considered a competing risk in statistical analyses. Outcomes were identified using International Classification of Diseases, 9th Revision, Clinical Modification (ICD-9-CM) and ICD-10 codes. Survival status, date of death, and cause of death were linked and verified using the Taiwan Death Registry.

Covariates included age, sex, left ventricular ejection fraction (LVEF), CHA₂DS₂-VASc score [congestive heart failure, hypertension, age ≥75 years (2 points), diabetes mellitus, prior stroke/transient ischemic attack/systemic embolism (2 points), vascular disease, age 65–74 years, and sex category], comorbidities, and medications. Comorbidities included hypertension, hyperlipidemia, diabetes mellitus, coronary artery disease, myocardial infarction, heart failure, atrial fibrillation, venous thromboembolism, cerebrovascular disease, peripheral artery disease, chronic obstructive pulmonary disease, autoimmune disease, chronic kidney disease, liver cirrhosis, and cancer. Medications included angiotensin-converting enzyme inhibitors, angiotensin receptor blockers, non-vitamin K antagonist oral anticoagulants (NOAC), warfarin, antiplatelet agents, beta-blockers, digoxin, non-dihydropyridine calcium channel blockers, statins, and oral hypoglycemic agents.

### Statistical analysis

Continuous variables were compared using analysis of variance, and categorical variables using the chi-square test. Control patients without thrombus were selected using a two-stage matching process. First, patients were matched 1:200 by sex, birth year, and echocardiography year to generate a control pool. Second, propensity matching up to 1:4 incorporated sex, age/birth year, echocardiography year, LVEF, CHA_2_DS_2_-VASc score, comorbidities, and medications. Covariate balance was assessed using absolute standardized mean differences. Clinical outcomes were compared using Cox proportional hazards models and are reported as hazard ratios (HRs) with 95% confidence intervals (CIs). A two-sided *p*–value <0.05 was considered nominally statistically significant. No formal adjustment for multiple testing was applied; because four chamber-specific groups were evaluated across six outcomes, these analyses were considered exploratory and hypothesis-generating. All statistical analyses were performed using SAS 9.4 (SAS Institute, Cary, NC, USA).

## Results

### Study population

Between 1 January 2001 and 31 December 2022, a total of 762,442 patients with echocardiographic records were screened in the Chang Gung Memorial Hospital System. Among them, 1,891 patients were identified as having intracardiac thrombus formation and were categorized by chamber before final propensity matching as LA (*n* = 763), LV (*n* = 951), RA (*n* = 143), or RV (*n* = 34) thrombus (Figure 1).

**Figure 1:**
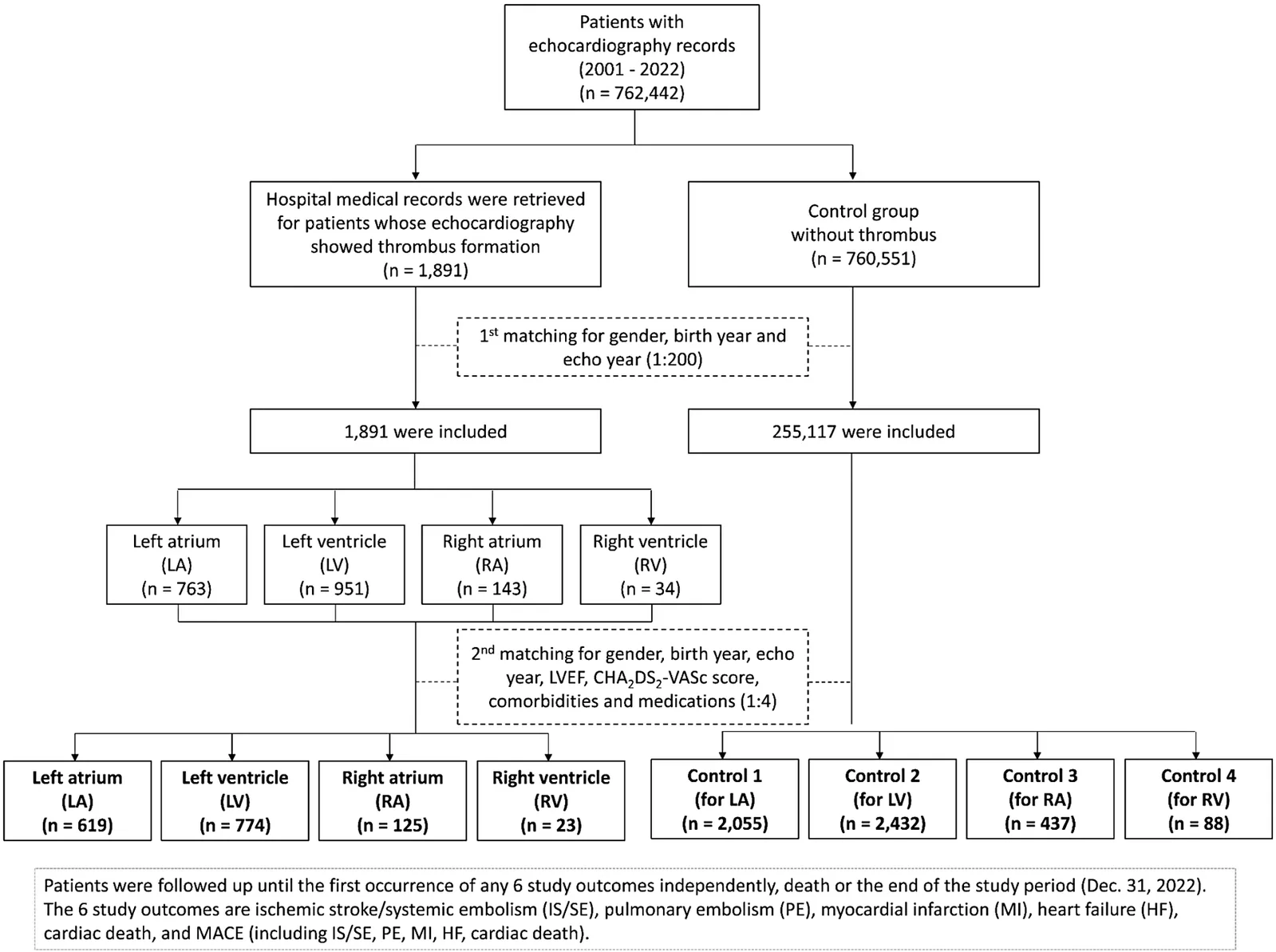
Study design and screening criteria flow chart for inclusion of patients with echocardiography-reported cardiac chamber thrombus and matched controls.

For each thrombus group, control patients without thrombus were selected from the remaining cohort using a two-step matching process. In the first step, patients were matched 1:200 by sex, birth year, and echocardiography year, yielding a control pool of 255,117 individuals. In the second step, patients were matched 1:4 additionally incorporating LVEF, CHA_2_DS_2_-VASc score, comorbidities, and medications. The final matched analytic cohorts comprised 619 patients with LA thrombus and 2,055 controls, 774 patients with LV thrombus and 2,432 controls, 125 patients with RA thrombus and 437 controls, and 23 patients with RV thrombus and 88 controls (Figure 1). Expanded matching substantially improved covariate balance across the chamber-specific cohorts, although residual imbalance remained for selected variables, particularly in the smaller RA and RV cohorts and for LVEF in the LV cohort (Supplementary Tables 1–4).

Patients were followed until the first occurrence of one of the predefined study outcomes, death, or the end of follow-up on 31 December 2022 (Figure 1). The six outcomes of interest were IS/SE, PE, MI, HF, cardiac death, and MACE. Baseline characteristics of the four chamber-specific cohorts and their matched controls are summarized in Supplementary Tables 1–4; baseline characteristics of patients with vs. without left atrial appendage (LAA) thrombus are shown in Supplementary Table 7. Mean follow-up durations after matching among thrombus patients were 5.67 ± 5.22 years for LA, 5.92 ± 5.50 years for LV, 3.73 ± 4.93 years for RA, and 3.81 ± 5.03 years for RV thrombus.

### Cardiovascular outcomes

#### LA thrombus

Compared with matched controls without LA thrombus, patients with LA thrombus had higher risks of IS/SE (HR: 1.85, 95% CI: 1.44–2.36; *p* < 0.0001), HF (HR: 1.30, 95% CI: 1.09–1.56; *p* = 0.0041), cardiac death (HR: 2.02, 95% CI: 1.55–2.64; *p* < 0.0001), and MACE (HR: 1.61, 95% CI: 1.39–1.86; *p* < 0.0001). No significant associations were observed for PE (HR: 1.45, 95% CI: 0.66–3.18; *p* = 0.3572) or MI (HR: 1.30, 95% CI: 0.77–2.19; *p* = 0.3288). Time-to-event curves were concordant, showing significant differences for IS/SE (*p* < 0.0001), HF (*p* = 0.0011), cardiac death (*p* < 0.0001), and MACE (*p* < 0.0001), but not PE (*p* = 0.3482) or MI (*p* = 0.3283) (Figure 2).

**Figure 2:**
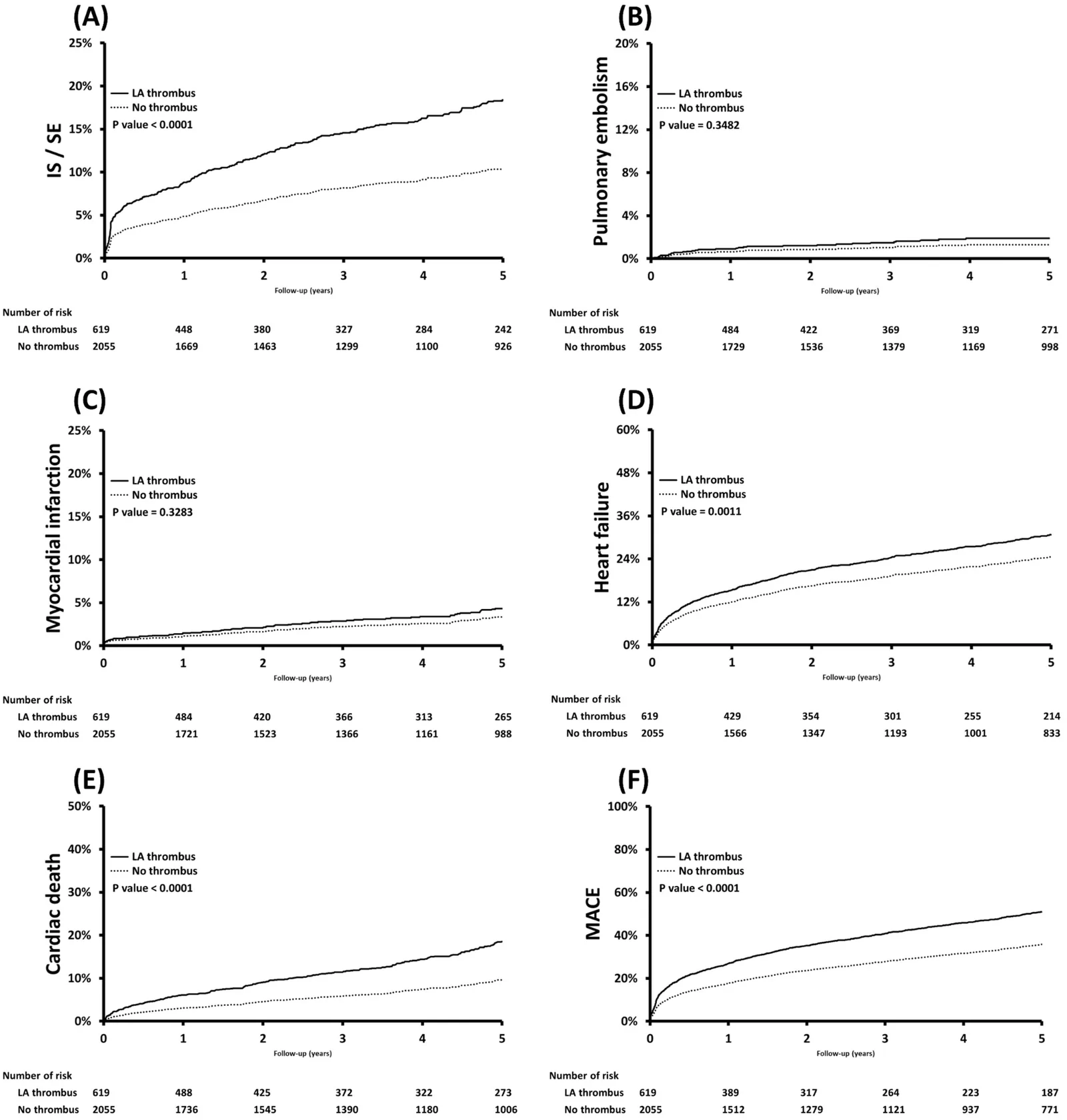
Time-to-event curves for patients with LA thrombus vs. matched controls without thrombus. **(A)** IS/SE, **(B)** pulmonary embolism, **(C)** myocardial infarction, **(D)** heart failure, **(E)** cardiac death, and **(F)** MACE.

#### LV thrombus

Compared with matched controls, LV thrombus was associated with increased risks of IS/SE (HR: 1.38, 95% CI: 1.05–1.80; *p* = 0.0201), MI (HR: 1.58, 95% CI: 1.23–2.04; *p* = 0.0004), HF (HR: 1.40, 95% CI: 1.22–1.60; *p* < 0.0001), cardiac death (HR: 1.32, 95% CI: 1.06–1.64; *p* = 0.0147), and MACE (HR: 1.36, 95% CI: 1.21–1.53; *p* < 0.0001). PE was not significantly increased (HR: 1.79, 95% CI: 0.66–4.82; *p* = 0.2505). Time-to-event analyses similarly showed differences for IS/SE (*p* = 0.0171), MI (*p* = 0.0003), HF (*p* < 0.0001), cardiac death (*p* = 0.0155), and MACE (*p* < 0.0001), but not PE (*p* = 0.2456) (Figure 3).

**Figure 3:**
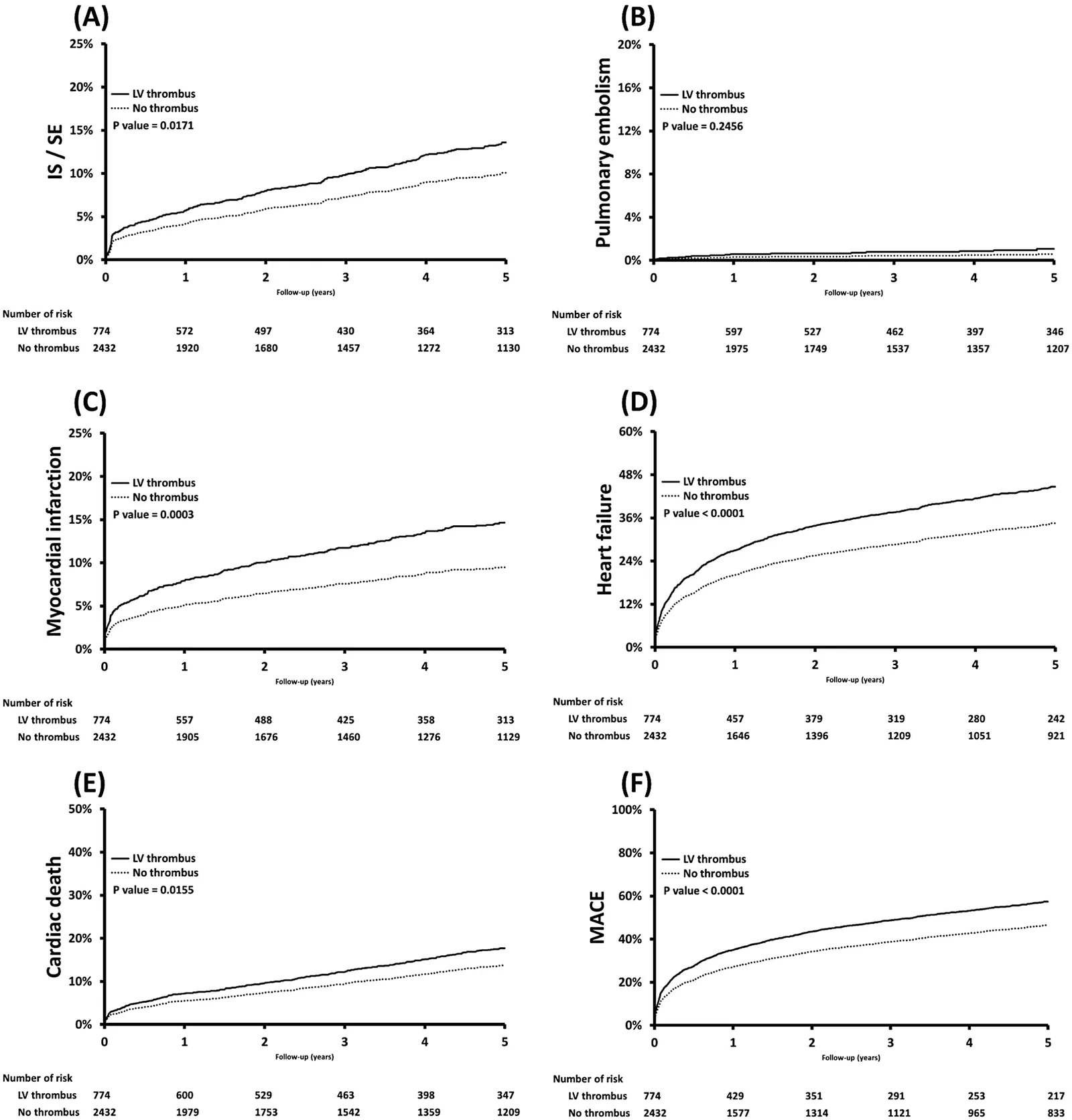
Time-to-event curves for patients with LV thrombus vs. matched controls without thrombus. **(A)** IS/SE, **(B)** pulmonary embolism, **(C)** myocardial infarction, **(D)** heart failure, **(E)** cardiac death, and **(F)** MACE.

#### RA thrombus

RA thrombus was associated with selective but pronounced risks. IS/SE (HR: 0.43, 95% CI: 0.13–1.42; *p* = 0.1666) and myocardial infarction (HR: 1.03, 95% CI: 0.22–4.78; *p* = 0.9714) did not differ significantly from controls. In contrast, pulmonary embolism (HR: 4.63, 95% CI: 1.79–11.98; *p* = 0.0016), heart failure (HR: 1.70, 95% CI: 1.01–2.85; *p* = 0.0440), and MACE (HR: 1.63, 95% CI: 1.08–2.45; *p* = 0.0198) were significantly increased. Cardiac death was not significantly increased (HR: 1.96, 95% CI: 0.84–4.61; *p* = 0.1220). Time-to-event analyses showed higher cumulative incidence of pulmonary embolism (*p* < 0.0001), heart failure (*p* < 0.0001), cardiac death (*p* = 0.0110), and MACE (*p* = 0.0005), but not IS/SE (*p* = 0.1639) or myocardial infarction (*p* = 0.4882) (Figure 4).

**Figure 4:**
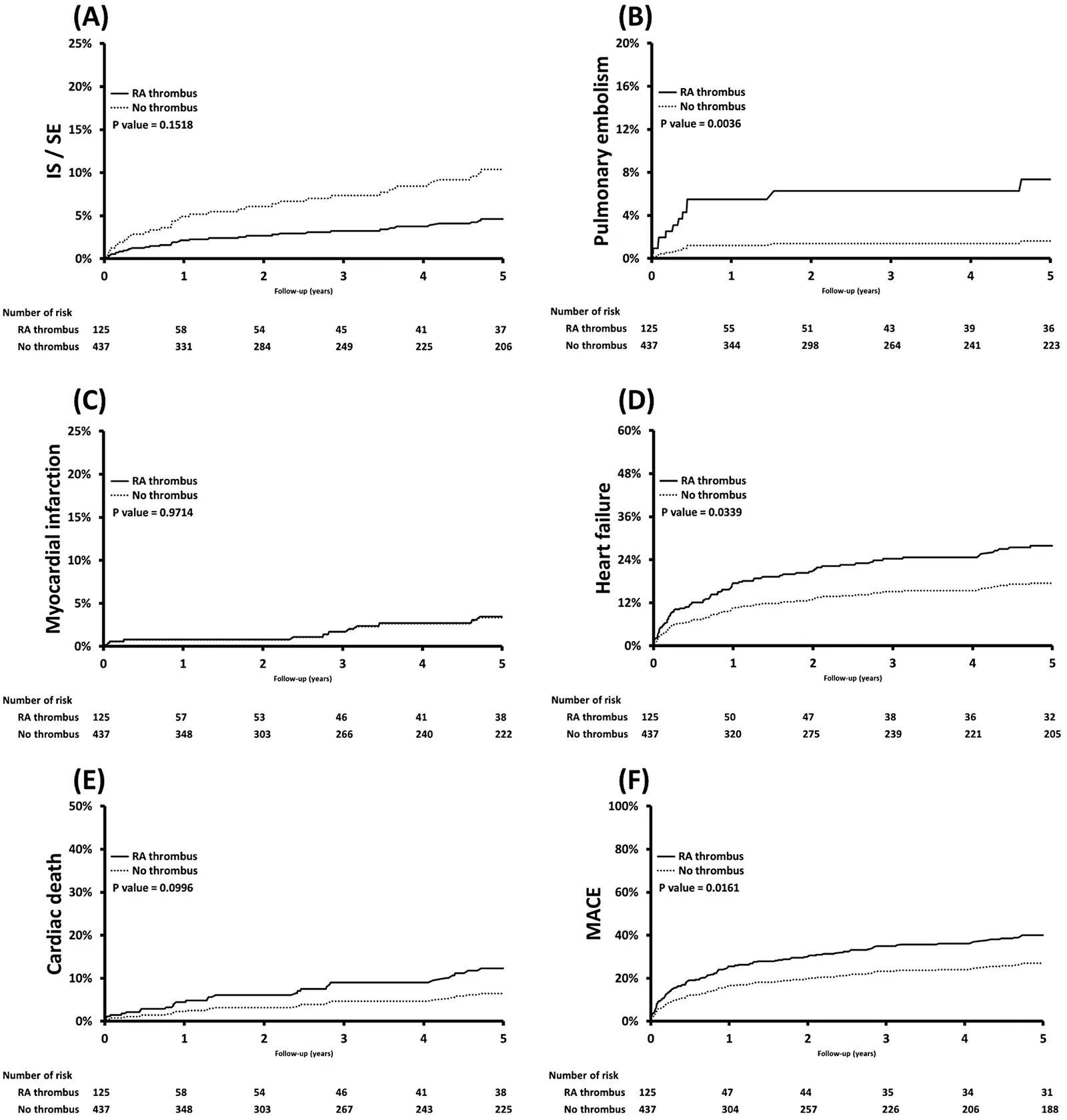
Time-to-event curves for patients with RA thrombus vs. matched controls without thrombus. **(A)** IS/SE, **(B)** pulmonary embolism, **(C)** myocardial infarction, **(D)** heart failure, **(E)** cardiac death, and **(F)** MACE.

#### RV thrombus

Prognostic interpretation of RV thrombus was limited by the very small sample size. (*n* = 23) and sparse events. No significant association was detected for IS/SE (HR: 0.88, 95% CI: 0.10–7.73; *p* = 0.9089), PE (HR: 1.88, 95% CI: 0.19–18.62; *p* = 0.5883), HF (HR: 1.49, 95% CI: 0.38–5.95; *p* = 0.5682), or MACE (HR: 1.31, 95% CI: 0.38–4.49; *p* = 0.6722). No MI or cardiac death events occurred in the RV thrombus group, precluding HR estimation. Time-to-event analyses similarly showed no significant differences for IS/SE (*p* = 0.9067), PE (*p* = 0.5774), HF (*p* = 0.5350), or MACE (*p* = 0.6332) (Figure 5). These findings should be interpreted as inconclusive rather than as evidence of absence of risk, because of limited statistical power. Clinical outcomes associated with thrombus in each cardiac chamber are summarized in Supplementary Table 5 and Figure 6.

**Figure 5:**
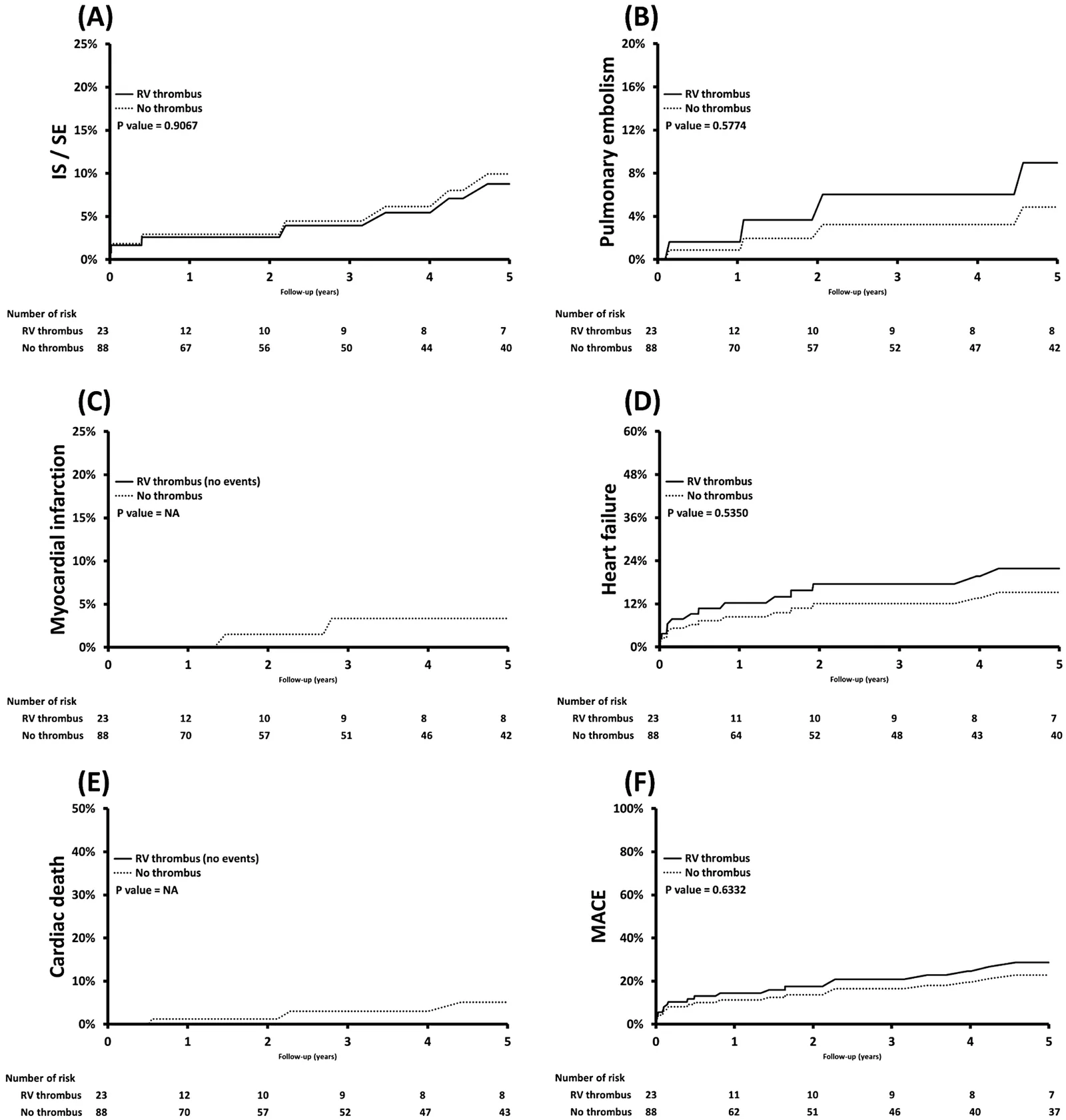
Time-to-event curves for patients with RV thrombus vs. matched controls without thrombus. **(A)** IS/SE, **(B)** pulmonary embolism, **(C)** myocardial infarction, **(D)** heart failure, **(E)** cardiac death, and **(F)** MACE.

**Figure 6:**
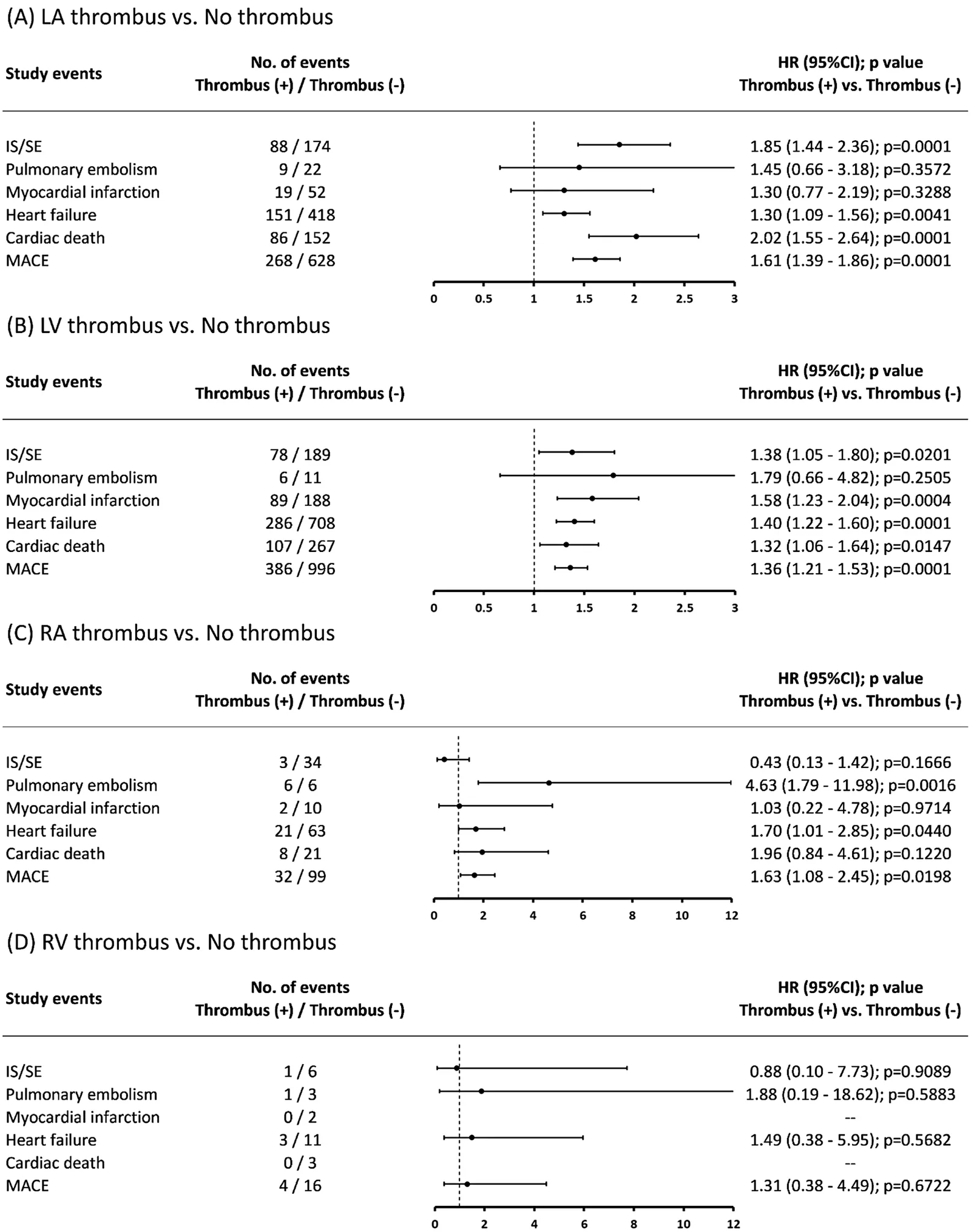
Cox proportional hazard ratios (95% confidence intervals) and *p*-values for cardiovascular outcomes according to cardiac chamber of the thrombus: (**A**) LA thrombus vs. no thrombus; (**B**) LV thrombus vs. no thrombus; (**C**) RA thrombus vs. no thrombus; and (**D**) RV thrombus vs. no thrombus.

#### Left- vs. right-heart thrombus

When grouped anatomically, left-heart thrombi (LA or LV) were associated with higher risk of IS/SE (HR: 1.59, 95% CI: 1.33–1.91; *p* < 0.0001), MI (HR: 1.54, 95% CI: 1.23–1.93; *p* = 0.0002), HF (HR: 1.37, 95% CI: 1.23–1.53; *p* < 0.0001), cardiac death (HR: 1.57, 95% CI: 1.33–1.86; *p* < 0.0001), and MACE (HR: 1.46, 95% CI: 1.33–1.60; *p* < 0.0001), with no significant association with PE (HR: 1.54, 95% CI: 0.83–2.85; *p* = 0.1685). Right-heart thrombi (RA or RV) were associated most strongly with PE (HR: 3.86, 95% CI: 1.64–9.09; *p* = 0.0020), with nominal associations with HF (HR: 1.67, 95% CI: 1.03–2.72; *p* = 0.0377) and MACE (HR: 1.59, 95% CI: 1.07–2.35; *p* = 0.0203). IS/SE, MI, and cardiac death were not significantly different from controls (Supplementary Table 6 and Figure 7).

**Figure 7:**
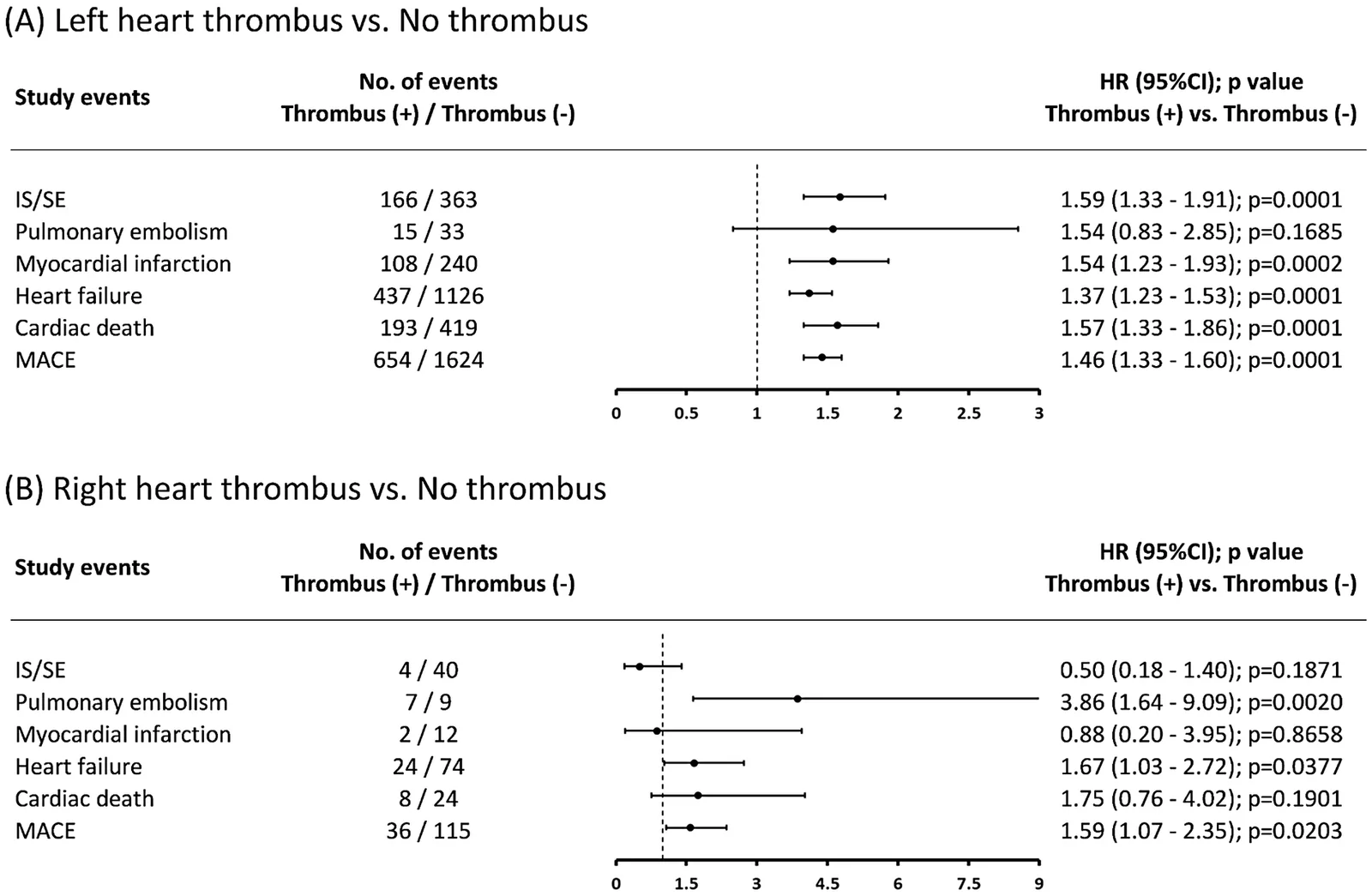
Cox proportional hazard ratios (95% confidence intervals) and *p*-values for cardiovascular outcomes according to thrombus location: (**A**) left-heart thrombus vs. no thrombus and (**B**) right-heart thrombus vs. no thrombus.

#### LAA thrombus

Dedicated analysis of LAA thrombus showed no incremental long-term risk compared with non-LAA LA thrombus after matching. Hazard ratios were neutral for IS/SE (HR: 0.88, 95% CI: 0.56–1.40; *p* = 0.5953), PE (HR: 0.96, 95% CI: 0.19–4.86; *p* = 0.9604), MI (HR: 0.85, 95% CI: 0.30–2.36; *p* = 0.7507), HF (HR: 1.04, 95% CI: 0.74–1.45; *p* = 0.8308), cardiac death (HR: 0.87, 95% CI: 0.56–1.35; *p* = 0.5254), and MACE (HR: 0.88, 95% CI: 0.67–1.14; *p* = 0.3246) (Supplementary Tables 7, 8, Figure 8).

**Figure 8:**
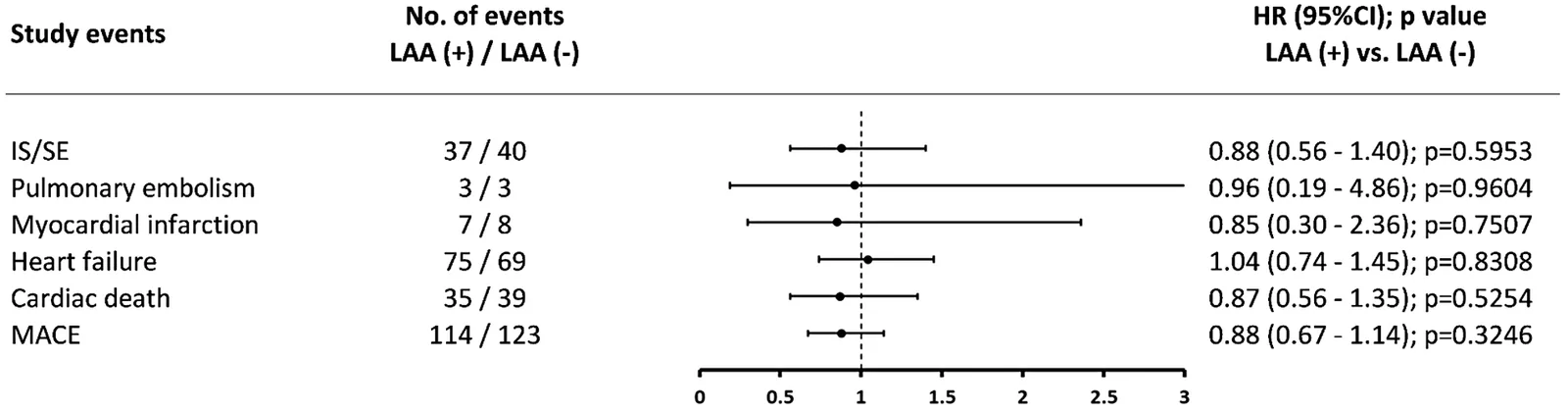
Cox proportional hazard ratios (95% confidence intervals) and *p*-values for cardiovascular outcomes according to the presence vs. absence of LAA thrombus among patients with LA thrombus.

## Discussion

To our knowledge, this is the first large-scale study to comprehensively compare long-term cardiovascular outcomes of echocardiography-detected intracardiac thrombus across all four cardiac chambers against matched controls. The revised analysis incorporated LVEF and CHA_2_DS_2_-VASc score into propensity matching, addressing important differences in cardiac function and baseline thromboembolic risk. Four findings emerge. First, LA and LV thrombi remained associated with multiple adverse cardiovascular outcomes after expanded matching. Second, RA thrombus showed a predominantly pulmonary embolic phenotype, with a strong association with PE, whereas cardiac death was not significantly associated after the more comprehensive matching. Third, no significant association was detected for RV thrombus, but the small subgroup and wide confidence intervals substantially limit inference. Fourth, LAA thrombus did not confer incremental long-term risk compared with non-LAA LA thrombus.

### Chamber-by-chamber perspective

#### LA thrombus

LA thrombus constituted a major subset of this cohort and remained associated with higher risks of IS/SE, HF, cardiac death, and MACE after matching that included LVEF and CHA_2_DS_2_-VASc score. This pattern is pathophysiologically compatible with atrial cardiomyopathy and AF-related stasis, in which impaired atrial transport and structural remodeling promote thrombogenesis and downstream embolic events. Importantly, LAA location did not confer incremental risk compared with non-LAA LA thrombus. This should not be interpreted as evidence that anatomic location is clinically irrelevant; rather, once LA thrombosis is present, long-term prognosis may depend more on the underlying atrial substrate—including AF burden, atrial remodeling, impaired atrial mechanical function, and systemic comorbidity—than on whether thrombus is confined to the appendage.

In patients with mitral stenosis who were in sinus rhythm, the incidence of LA thrombus was 6.6% (17). TEE is indicated in patients with an LA infero-superior dimension >6.9 cm and mean mitral gradient >18 mmHg (17). In addition, when spontaneous echo contrast (SEC) is absent on echo exam, thrombus formation is unlikely (17). When the LA appendage ejection velocity is <0.4 m/s, measured using pulsed-wave Doppler 1 cm into the opening of the appendage, there is an increased risk of thrombus formation (18). In another study, independent predictors for the presence of thrombus or dense SEC included ejection fraction <40% and LA diameter ≥50 mm (19). In anticoagulated patients, thrombus and dense SEC were significantly associated with an increased risk for stroke or death in 5 years of follow-up (OR: 1.55, 95% CI: 0.50–4.83) (19).

#### LV thrombus

LV thrombus remained associated with a broad cardiovascular risk profile after additional matching for LVEF and CHA_2_DS_2_-VASc score, including increased risks of IS/SE, MI, HF, cardiac death, and MACE. These findings extend the established relationship between LV thrombus and embolic events by demonstrating persistent long-term risk beyond the acute infarction setting. Contemporary multimodality imaging studies continue to emphasize the importance of LVEF and apical dysfunction in LV thrombus formation, and the greater sensitivity of cardiac magnetic resonance compared with routine TTE for thrombus detection (20, 21).

LV thrombus formation was typically found attached to areas of poor LV wall motion. Echocardiography is most commonly used modality in detecting LV thrombi visually adjacent to wall motion abnormality, when suspicion is high (22). Occasionally, echocardiography with contrast opacification, computed tomography, or cardiac magnetic resonance imaging may be necessary to identify thrombus with greater confidence and accuracy (23). Previous studies identified two major echocardiographic risk factors for embolization: thrombus mobility and thrombus protrusion (24–27). In one report, embolization occurred in 22% of 119 patients with an LV thrombus after acute myocardial infarction (24). Among 18 patients with free thrombus mobility, embolization occurred in 15 (83%) (24). In another study, among the 40 patients with thrombus protrusion, embolization occurred in 23 (58%) (25).

#### RA thrombus

Although less frequent, RA thrombus exhibited disproportionate risk—particularly for pulmonary embolism, heart failure, and cardiac death—echoing the lethality described for clot-in-transit. The present matched, longitudinal analysis extends beyond the acute setting and supports codified care pathways that prioritize rapid recognition, prompt anticoagulation, and consideration of advanced therapies when hemodynamic instability is present.

In setting of atrial fibrillation or atrial flutter, flow disturbance and the consequent thrombogenicity in the RA also predispose to thrombus formation. A Swedish study reported that from 23,796 autopsies, there was a prevalence of 7% of RA thrombus, which was similar to the prevalence of LA thrombus (28). In addition, RA thrombus has been described as the cause of about 10% of pulmonary embolism cases (28). Among patients with RA thrombus, 36% had pulmonary embolism and 6.5% of all patients with pulmonary embolism were confirmed at autopsy to have RA thrombus (28).

#### RV thrombus

RV thrombus was rare in this cohort and showed no significant long-term signal after matching. This observation aligns with heterogeneous findings from small series and underscores the need for larger multicenter efforts to define its true incidence and optimal management. Rare cardiomyopathies predominantly involving the RV, such as the arrhythmogenic right ventricular cardiomyopathy (ARVC), have been associated with RV thrombus formation (29). In patients with Loeffler endocarditis, a subtype of idiopathic hypereosinophilic syndrome, the myocardium is infiltrated by white blood cells, resulting in thickening of heart muscle (30). This in turn can lead to biventricular restrictive physiology, which is a rare cause of RV mural and apical thrombus formation, and may trigger fatal ventricular fibrillation (30). The consequences of RV thrombus may be pulmonary embolism with decreased preload and hypotension in these patients. In a study of 138 patients (69 males) aged 62 ± 19 years with RV thrombus, pulmonary embolism mortality was higher in 31 patients with systolic blood pressure <90 mmHg than in 107 normotensives (42% vs. 12%, *p* = 0.0002) (31).

#### Comparative risks across cardiac chambers

These results highlight the strongly location-dependent prognostic impact of intracardiac thrombus. Cox proportional hazards models demonstrated that LA and LV thrombi conferred the broadest adverse cardiovascular outcomes, with significantly increased risks of ischemic stroke/systemic embolism, myocardial infarction, heart failure, cardiac death, and MACE. In contrast, RA thrombus exhibited a more selective yet pronounced risk profile, driven primarily by heart failure, cardiac death, and MACE, without a corresponding increase in left-sided embolic events. RV thrombus, although rare, showed no meaningful long-term association with any of the prespecified outcomes. Overall, these findings underscore that the clinical consequences of intracardiac thrombus are not uniform but are heavily determined by the chamber involved.

#### Left-heart thrombus vs. right-heart thrombus

Aggregating by anatomical side clarifies the biology. Left-sided thrombi (LA/LV) are linked to systemic embolization and downstream cardiac events—including myocardial infarction, heart failure, and mortality—while right-sided thrombi (RA/RV) align with pulmonary embolism and hemodynamic compromise. The current analysis quantifies this side-specific pattern across chambers with matched comparators and comprehensive endpoints, strengthening the rationale for location-specific clinical pathways.

#### Presence of LAA thrombus

Taken together, these data fill an important gap by providing a head-to-head, chamber-resolved comparison of long-term outcomes against matched controls and across comprehensive endpoints. LV and LA thrombi emerge as the most clinically consequential; RA thrombus is less common but clearly mortality-prone; and RV thrombus is rare with limited long-term impact. Notably, LAA thrombus does not add prognostic burden beyond non-LAA LA thrombus. Collectively, these results support location-specific risk stratification, anticoagulation strategies, and surveillance pathways in patients with intracardiac thrombus.

In this multi-institutional cohort spanning 2001–2022 and incorporating both transthoracic and transesophageal echocardiography, we deliver a chamber-resolved map of long-term risk for intracardiac thrombi against rigorously matched non-thrombus controls. Three findings stand out. First, prognosis is location-dependent, with LV and LA thrombi conferring the broadest cardiovascular risk. Second, RA thrombus—although less common—is disproportionately associated with pulmonary embolism and cardiac death. Third, RV thrombus is rare and did not significantly predict adverse outcomes, likely reflecting both low incidence and sparse events. In addition, LAA thrombus did not carry incremental risk compared with non-LAA LA thrombus, indicating that long-term prognosis hinges on the presence of LA thrombus itself rather than appendage confinement.

The present results confirm and extend prior observations for LV thrombus—classically described after myocardial infarction—by demonstrating independent associations with stroke or systemic embolism, myocardial infarction, heart failure, cardiac death, and MACE in a real-world echocardiographic population rather than infarct-only cohorts. For LA/LAA thrombus, prior studies in AF populations emphasized pre-procedural TEE prevalence and short-term peri-procedural risk; our data move beyond prevalence to quantify matched, long-term hazards for LA thrombus and add the clinically relevant finding that LAA location does not worsen prognosis relative to non-LAA sites. For right-sided thrombi, the existing literature is dominated by PE-linked series and meta-analyses of clot-in-transit, highlighting high short-term mortality and heterogeneous treatment. By disentangling RA from RV thrombus and quantifying long-term outcomes against matched controls, the present study complements that literature: RA thrombus tracks with pulmonary embolism, heart failure, cardiac death, and MACE, whereas RV thrombus is rare with limited signal outside the hyperacute phase.

The chamber-resolved risk profiles observed here have direct clinical relevance. Patients with LV or LA thrombus warrant intensified follow-up and secondary prevention given broad, durable risks spanning thromboembolic and heart failure endpoints; decisions regarding the intensity and duration of anticoagulation should be individualized to ischemic and bleeding risk and informed by serial imaging. RA thrombus should trigger a pulmonary embolism-centric management lens, emphasizing rapid anticoagulation and timely consideration of advanced therapies in unstable patients. Within the LA, the lack of incremental risk for LAA vs. non-LAA thrombus indicates that management should be driven by the presence of LA thrombus *per se*, with future risk models incorporating LA function, AF burden, and heart failure status rather than appendage location alone. Finally, when echocardiography is equivocal and the downstream therapeutic pathway would change, a selective cardiac magnetic resonance (CMR) strategy may improve diagnostic certainty.

## Limitations

This study has several limitations. First, case identification relied in part on administrative coding (ICD-9-CM/ICD-10), which may introduce misclassification or under-ascertainment. Second, echocardiographic assessments (TTE and TEE) were performed in routine clinical practice without centralized core laboratory adjudication; therefore, interobserver variability and lack of standardized evaluation of thrombus characteristics (e.g., size, mobility, and protrusion) may affect classification and risk estimation. Third, the number of patients with right ventricular thrombus was small with limited events, reducing statistical power and rendering these findings exploratory.

Fourth, although medication categories were captured, detailed treatment information—including adherence, dosing, anticoagulation intensity (e.g., time in therapeutic range), and longitudinal treatment changes—was unavailable, precluding assessment of treatment effects. Fifth, despite rigorous propensity score matching across demographics, comorbidities, and medications, residual confounding from unmeasured variables (e.g., ventricular function, atrial fibrillation burden, valvular disease severity, and thrombus morphology) cannot be excluded.

Finally, the cohort was derived from a large tertiary-care healthcare system in Taiwan, which may introduce referral bias and limit generalizability to other healthcare settings or populations; external validation in diverse cohorts is warranted.

## Conclusions

The prognostic impact of intracardiac thrombus is strongly location-dependent. LA and LV thrombi were associated with the broadest adverse cardiovascular risks, whereas RA thrombus was more selectively associated with pulmonary embolism and cardiac mortality. RV thrombus was uncommon and showed no significant long-term prognostic signal, although interpretation is limited by small numbers. LAA thrombus did not confer incremental risk beyond non-LAA LA thrombus. These findings support chamber-specific risk stratification and management strategies.

## Data Availability

The data are available upon reasonable request to the corresponding author but are not publicly available due to ethical and privacy restrictions. Access requires approval from the Chang Gung Memorial Hospital research ethics committee.
