# Supplementary material for: Chamber-specific long-term prognosis of intracardiac thrombus: a 21-year propensity-matched cohort study
Source: Front Cardiovasc Med. 2026 Oct 13;13:1889091. doi: 10.3389/fcvm.2026.1889091 (PMC13710267; doi:10.3389/fcvm.2026.1889091)
Supplement: Supplementary file 1 [file Table1.docx]

**Supplemental Tables**

**Supplemental Table 1.** Baseline characteristics of study patients with left atrial (LA) thrombus

**Supplemental Table 2.** Baseline characteristics of study patients with left ventricular (LV) thrombus

**Supplemental Table 3.** Baseline characteristics of study patients with right atrial (RA) thrombus

**Supplemental Table 4.** Baseline characteristics of study patients with right ventricular (RV) thrombus

**Supplemental Table 5.** Incidence rate of cardiovascular outcomes according to cardiac chamber of the thrombus

**Supplemental Table 6.** Incidence rate of cardiovascular outcomes according to left heart thrombus and right heart thrombus

**Supplemental Table 7.** Baseline characteristics of study patients with left atrial appendage (LAA) vs. without LAA

**Supplemental Table 8.** Incidence rate of cardiovascular outcomes according to left atrial appendage (LAA) thrombus (+) and LAA(-)

**Supplemental Table 1.** Baseline characteristics of study patients with left atrial (LA) thrombus

|  | Before matching | | | | |  | After matching | | | | |
| --- | --- | --- | --- | --- | --- | --- | --- | --- | --- | --- | --- |
|  | LA thrombus | | No thrombus | | ASMD |  | LA thrombus | | No thrombus | | ASMD |
|  | (n = 763) | | (n = 147,345) | |  |  | (n= 619) | | (n = 2,055) | |  |
| Age, mean ± SD | 64.85 | 13.81 | 64.28 | 12.49 | 0.0431 |  | 66.09 | 13.47 | 66.94 | 13.31 | 0.0632 |
| Charlson Comorbidity Index, mean ± SD | 2.62 | 2.16 | 2.38 | 2.29 | 0.1063 |  | 2.6 | 2.19 | 2.75 | 2.31 | 0.0693 |
| LVEF, mean ± SD | 59.05 | 14.79 | 66.67 | 12.13 | 0.5638 |  | 59.94 | 14.76 | 60.12 | 14.89 | 0.0126 |
| CHA₂DS₂-VASc score, mean ± SD | 3.2 | 1.95 | 2.54 | 1.79 | 0.3510 |  | 3.25 | 1.97 | 3.26 | 1.98 | 0.0063 |
| Male | 373 | 48.89% | 78223 | 53.09% | 0.0841 |  | 306 | 49.43% | 1031 | 50.17% | 0.0147 |
| Comorbidities |  |  |  |  |  |  |  |  |  |  |  |
| Hypertension | 385 | 50.46% | 84432 | 57.30% | 0.1376 |  | 335 | 54.12% | 1167 | 56.79% | 0.0537 |
| Hyperlipidemia | 247 | 32.37% | 49836 | 33.82% | 0.0308 |  | 199 | 32.15% | 676 | 32.90% | 0.0159 |
| Diabetes mellitus | 176 | 23.07% | 40880 | 27.74% | 0.1076 |  | 141 | 22.78% | 502 | 24.43% | 0.0389 |
| Coronary artery disease | 193 | 25.29% | 46290 | 31.42% | 0.1361 |  | 162 | 26.17% | 554 | 26.96% | 0.0178 |
| Myocardial infarction | 32 | 4.19% | 9781 | 6.64% | 0.1081 |  | 27 | 4.36% | 113 | 5.50% | 0.0525 |
| Congestive heart failure | 365 | 47.84% | 26690 | 18.11% | 0.6664 |  | 275 | 44.43% | 866 | 42.14% | 0.0461 |
| Atrial fibrillation | 549 | 71.95% | 12525 | 8.50% | 1.6971 |  | 419 | 67.69% | 1331 | 64.77% | 0.0618 |
| Venous thromboembolism | 15 | 1.97% | 1221 | 0.83% | 0.0970 |  | 12 | 1.94% | 34 | 1.65% | 0.0214 |
| Cerebrovascular disease | 290 | 38.01% | 27055 | 18.36% | 0.4475 |  | 228 | 36.83% | 735 | 35.77% | 0.0222 |
| Peripheral artery disease | 16 | 2.10% | 1695 | 1.15% | 0.0750 |  | 13 | 2.10% | 37 | 1.80% | 0.0217 |
| Chronic obstructive pulmonary disease | 148 | 19.40% | 24822 | 16.85% | 0.0663 |  | 120 | 19.39% | 436 | 21.22% | 0.0455 |
| Autoimmune disease | 21 | 2.75% | 4747 | 3.22% | 0.0276 |  | 17 | 2.75% | 68 | 3.31% | 0.0328 |
| Chronic kidney disease | 158 | 20.71% | 26182 | 17.77% | 0.0746 |  | 129 | 20.84% | 489 | 23.80% | 0.0710 |
| Liver Cirrhosis | 8 | 1.05% | 1617 | 1.10% | 0.0047 |  | 7 | 1.13% | 20 | 0.97% | 0.0154 |
| Cancer | 60 | 7.86% | 23121 | 15.69% | 0.2447 |  | 54 | 8.72% | 186 | 9.05% | 0.0115 |
| Medications |  |  |  |  |  |  |  |  |  |  |  |
| ACEi | 121 | 15.86% | 17482 | 11.86% | 0.1158 |  | 97 | 15.67% | 300 | 14.60% | 0.0299 |
| ARB | 250 | 32.77% | 41650 | 28.27% | 0.0978 |  | 208 | 33.60% | 703 | 34.21% | 0.0128 |
| Direct oral anticoagulant | 112 | 14.68% | 3084 | 2.09% | 0.4662 |  | 88 | 14.22% | 300 | 14.60% | 0.0109 |
| Warfarin | 340 | 44.56% | 4343 | 2.95% | 1.1209 |  | 232 | 37.48% | 582 | 28.32% | 0.1959 |
| Antiplatelet | 320 | 41.94% | 50166 | 34.05% | 0.1632 |  | 268 | 43.30% | 913 | 44.43% | 0.0228 |
| Beta blocker | 179 | 23.46% | 26454 | 17.95% | 0.1362 |  | 133 | 21.49% | 459 | 22.34% | 0.0205 |
| Digoxin | 279 | 36.57% | 5499 | 3.73% | 0.8972 |  | 204 | 32.96% | 520 | 25.30% | 0.1690 |
| Nondihydropyridine CCB | 157 | 20.58% | 11541 | 7.83% | 0.3713 |  | 128 | 20.68% | 369 | 17.96% | 0.0690 |
| Statin | 153 | 20.05% | 31242 | 21.20% | 0.0284 |  | 129 | 20.84% | 471 | 22.92% | 0.0503 |
| Oral hypoglycemic agent | 108 | 14.15% | 21884 | 14.85% | 0.0198 |  | 81 | 13.09% | 288 | 14.01% | 0.0271 |
| Follow-up (years), mean ± SD | 5.89 | 5.19 | 7.07 | 5.30 |  |  | 5.67 | 5.22 | 6.12 | 5.08 |  |

ACEi, angiotensin converting enzyme inhibitor; ARB, angiotensin receptor blocker; ASMD, absolute standardized mean difference; CCB, calcium channel blocker; SD standard deviation

**Supplemental Table 2.** Baseline characteristics of study patients with left ventricular (LV) thrombus

|  | Before matching | | | | |  | After matching | | | | |
| --- | --- | --- | --- | --- | --- | --- | --- | --- | --- | --- | --- |
|  | LV thrombus | | No thrombus | | ASMD |  | LV thrombus | | No thrombus | | ASMD |
|  | (n = 951) | | (n = 165,602) | |  |  | (n= 774) | | (n = 2,432) | |  |
| Age, mean ± SD | 61.11 | 13.7 | 61.65 | 12.77 | 0.0406 |  | 61.96 | 12.8 | 62.32 | 12.4 | 0.0288 |
| Charlson Comorbidity Index, mean ± SD | 3.14 | 2.35 | 2.27 | 2.23 | 0.3802 |  | 3.03 | 2.32 | 3.04 | 2.28 | 0.0059 |
| LVEF, mean ± SD | 38.76 | 14.09 | 65.86 | 12.55 | 2.0310 |  | 41.01 | 13.68 | 43.98 | 15.3 | 0.2046 |
| CHA₂DS₂-VASc score, mean ± SD | 3.25 | 1.86 | 2.16 | 1.75 | 0.6049 |  | 3.17 | 1.83 | 3.15 | 1.87 | 0.0133 |
| Male | 763 | 80.23% | 128441 | 77.56% | 0.0655 |  | 634 | 81.91% | 2024 | 83.22% | 0.0346 |
| Comorbidities |  |  |  |  |  |  |  |  |  |  |  |
| Hypertension | 538 | 56.57% | 91242 | 55.10% | 0.0297 |  | 434 | 56.07% | 1428 | 58.72% | 0.0535 |
| Hyperlipidemia | 467 | 49.11% | 54087 | 32.66% | 0.3393 |  | 367 | 47.42% | 1173 | 48.23% | 0.0163 |
| Diabetes mellitus | 348 | 36.59% | 43601 | 26.33% | 0.2224 |  | 285 | 36.82% | 915 | 37.62% | 0.0166 |
| Coronary artery disease | 673 | 70.77% | 54538 | 32.93% | 0.8181 |  | 533 | 68.86% | 1658 | 68.17% | 0.0148 |
| Myocardial infarction | 482 | 50.68% | 13178 | 7.96% | 1.0628 |  | 361 | 46.64% | 1055 | 43.38% | 0.0656 |
| Congestive heart failure | 593 | 62.36% | 29717 | 17.94% | 1.0162 |  | 450 | 58.14% | 1359 | 55.88% | 0.0457 |
| Atrial fibrillation | 93 | 9.78% | 13574 | 8.20% | 0.0553 |  | 84 | 10.85% | 253 | 10.40% | 0.0146 |
| Venous thromboembolism | 6 | 0.63% | 1182 | 0.71% | 0.0101 |  | 5 | 0.65% | 13 | 0.53% | 0.0146 |
| Cerebrovascular disease | 230 | 24.19% | 29432 | 17.77% | 0.1580 |  | 171 | 22.09% | 585 | 24.05% | 0.0466 |
| Peripheral artery disease | 16 | 1.68% | 1854 | 1.12% | 0.0479 |  | 11 | 1.42% | 33 | 1.36% | 0.0055 |
| Chronic obstructive pulmonary disease | 170 | 17.88% | 27540 | 16.63% | 0.0330 |  | 130 | 16.80% | 451 | 18.54% | 0.0459 |
| Autoimmune disease | 31 | 3.26% | 4602 | 2.78% | 0.0281 |  | 24 | 3.10% | 83 | 3.41% | 0.0176 |
| Chronic kidney disease | 229 | 24.08% | 27394 | 16.54% | 0.1882 |  | 183 | 23.64% | 563 | 23.15% | 0.0117 |
| Liver Cirrhosis | 4 | 0.42% | 2124 | 1.28% | 0.0939 |  | 3 | 0.39% | 9 | 0.37% | 0.0029 |
| Cancer | 77 | 8.10% | 24451 | 14.76% | 0.2107 |  | 63 | 8.14% | 184 | 7.57% | 0.0213 |
| Medications |  |  |  |  |  |  |  |  |  |  |  |
| ACEi | 341 | 35.86% | 21649 | 13.07% | 0.5497 |  | 270 | 34.88% | 794 | 32.65% | 0.0473 |
| ARB | 421 | 44.27% | 45450 | 27.45% | 0.3563 |  | 326 | 42.12% | 1007 | 41.41% | 0.0145 |
| Direct oral anticoagulant | 39 | 4.10% | 2997 | 1.81% | 0.1356 |  | 29 | 3.75% | 89 | 3.66% | 0.0046 |
| Warfarin | 134 | 14.09% | 4898 | 2.96% | 0.4068 |  | 62 | 8.01% | 210 | 8.63% | 0.0226 |
| Antiplatelet | 720 | 75.71% | 60481 | 36.52% | 0.8595 |  | 576 | 74.42% | 1789 | 73.56% | 0.0196 |
| Beta blocker | 342 | 35.96% | 30688 | 18.53% | 0.3992 |  | 260 | 33.59% | 805 | 33.10% | 0.0104 |
| Digoxin | 120 | 12.62% | 6514 | 3.93% | 0.3192 |  | 84 | 10.85% | 270 | 11.10% | 0.0080 |
| Nondihydropyridine CCB | 80 | 8.41% | 13232 | 7.99% | 0.0154 |  | 67 | 8.66% | 210 | 8.63% | 0.0008 |
| Statin | 433 | 45.53% | 34922 | 21.09% | 0.5370 |  | 340 | 43.93% | 1042 | 42.85% | 0.0218 |
| Oral hypoglycemic agent | 204 | 21.45% | 23615 | 14.26% | 0.1886 |  | 164 | 21.19% | 491 | 20.19% | 0.0247 |
| Follow-up (years), mean ± SD | 5.77 | 5.39 | 7.21 | 5.53 |  |  | 5.92 | 5.50 | 6.51 | 5.66 |  |

ACEi, angiotensin converting enzyme inhibitor; ARB, angiotensin receptor blocker; ASMD, absolute standardized mean difference; CCB, calcium channel blocker; SD standard deviation

**Supplemental Table 3.** Baseline characteristics of study patients with right atrial (RA) thrombus

|  | Before matching | | | | |  | After matching | | | | |
| --- | --- | --- | --- | --- | --- | --- | --- | --- | --- | --- | --- |
|  | RA thrombus | | No thrombus | | ASMD |  | RA thrombus | | No thrombus | | ASMD |
|  | (n = 143) | | (n = 36,345) | |  |  | (n= 125) | | (n = 437) | |  |
| Age, mean ± SD | 63.41 | 15.47 | 64.23 | 12.99 | 0.0581 |  | 64.33 | 14.55 | 65.51 | 13.65 | 0.0840 |
| Charlson Comorbidity Index, mean ± SD | 3.59 | 2.6 | 2.34 | 2.26 | 0.5128 |  | 3.59 | 2.6 | 3.43 | 2.64 | 0.0609 |
| LVEF, mean ± SD | 61.29 | 16.35 | 66.5 | 12.21 | 0.3616 |  | 61.61 | 15.83 | 62.29 | 13.61 | 0.0462 |
| CHA₂DS₂-VASc score, mean ± SD | 2.94 | 2.05 | 2.53 | 1.82 | 0.2142 |  | 2.91 | 2.06 | 2.86 | 1.92 | 0.0236 |
| Male | 80 | 55.94% | 21239 | 57.96% | 0.0407 |  | 68 | 54.40% | 248 | 56.75% | 0.0473 |
| Comorbidities |  |  |  |  |  |  |  |  |  |  |  |
| Hypertension | 66 | 46.15% | 20796 | 56.75% | 0.2132 |  | 60 | 48.00% | 226 | 51.72% | 0.0744 |
| Hyperlipidemia | 33 | 23.08% | 11895 | 32.46% | 0.2107 |  | 31 | 24.80% | 121 | 27.69% | 0.0657 |
| Diabetes mellitus | 41 | 28.67% | 10154 | 27.71% | 0.0214 |  | 38 | 30.40% | 145 | 33.18% | 0.0597 |
| Coronary artery disease | 35 | 24.48% | 11569 | 31.57% | 0.1585 |  | 33 | 26.40% | 138 | 31.58% | 0.1143 |
| Myocardial infarction | 11 | 7.69% | 2418 | 6.60% | 0.0425 |  | 9 | 7.20% | 39 | 8.92% | 0.0634 |
| Congestive heart failure | 48 | 33.57% | 6541 | 17.85% | 0.3656 |  | 40 | 32.00% | 126 | 28.83% | 0.0689 |
| Atrial fibrillation | 27 | 18.88% | 3006 | 8.20% | 0.3159 |  | 24 | 19.20% | 60 | 13.73% | 0.1479 |
| Venous thromboembolism | 15 | 10.49% | 244 | 0.67% | 0.4382 |  | 6 | 4.80% | 18 | 4.12% | 0.0330 |
| Cerebrovascular disease | 34 | 23.78% | 6765 | 18.46% | 0.1305 |  | 30 | 24.00% | 86 | 19.68% | 0.1047 |
| Peripheral artery disease | 2 | 1.40% | 381 | 1.04% | 0.0327 |  | 2 | 1.60% | 7 | 1.60% | 0.0001 |
| Chronic obstructive pulmonary disease | 22 | 15.38% | 6087 | 16.61% | 0.0335 |  | 20 | 16.00% | 81 | 18.54% | 0.0671 |
| Autoimmune disease | 5 | 3.50% | 1070 | 2.92% | 0.0327 |  | 3 | 2.40% | 12 | 2.75% | 0.0219 |
| Chronic kidney disease | 37 | 25.87% | 6178 | 16.86% | 0.2213 |  | 34 | 27.20% | 117 | 26.77% | 0.0096 |
| Liver Cirrhosis | 4 | 2.80% | 442 | 1.21% | 0.1138 |  | 3 | 2.40% | 13 | 2.97% | 0.0356 |
| Cancer | 54 | 37.76% | 5660 | 15.45% | 0.5220 |  | 47 | 37.60% | 158 | 36.16% | 0.0299 |
| Medications |  |  |  |  |  |  |  |  |  |  |  |
| ACEi | 28 | 19.58% | 4347 | 11.86% | 0.2132 |  | 23 | 18.40% | 86 | 19.68% | 0.0326 |
| ARB | 41 | 28.67% | 10362 | 28.28% | 0.0087 |  | 37 | 29.60% | 114 | 26.09% | 0.0784 |
| Direct oral anticoagulant | 9 | 6.29% | 638 | 1.74% | 0.2334 |  | 4 | 3.20% | 21 | 4.81% | 0.0820 |
| Warfarin | 29 | 20.28% | 924 | 2.52% | 0.5819 |  | 20 | 16.00% | 36 | 8.24% | 0.2395 |
| Antiplatelet | 59 | 41.26% | 12881 | 35.15% | 0.1260 |  | 50 | 40.00% | 190 | 43.48% | 0.0706 |
| Beta blocker | 30 | 20.98% | 6646 | 18.14% | 0.0717 |  | 25 | 20.00% | 85 | 19.45% | 0.0138 |
| Digoxin | 23 | 16.08% | 1356 | 3.70% | 0.4240 |  | 17 | 13.60% | 50 | 11.44% | 0.0653 |
| Nondihydropyridine CCB | 18 | 12.59% | 2823 | 7.70% | 0.1623 |  | 15 | 12.00% | 42 | 9.61% | 0.0770 |
| Statin | 18 | 12.59% | 7527 | 20.54% | 0.2152 |  | 16 | 12.80% | 50 | 11.44% | 0.0416 |
| Oral hypoglycemic agent | 23 | 16.08% | 5571 | 15.20% | 0.0243 |  | 22 | 17.60% | 77 | 17.62% | 0.0005 |
| Follow-up (years), mean ± SD | 3.67 | 4.78 | 7.05 | 5.11 |  |  | 3.73 | 4.93 | 6.38 | 5.52 |  |

ACEi, angiotensin converting enzyme inhibitor; ARB, angiotensin receptor blocker; ASMD, absolute standardized mean difference; CCB, calcium channel blocker; SD standard deviation

**Supplemental Table 4.** Baseline characteristics of study patients with right ventricular (RV) thrombus

|  | Before matching | | | | |  | After matching | | | | |
| --- | --- | --- | --- | --- | --- | --- | --- | --- | --- | --- | --- |
|  | RV thrombus | | No thrombus | | ASMD |  | RV thrombus | | No thrombus | | ASMD |
|  | (n = 34) | | (n = 7,596) | |  |  | (n= 23) | | (n = 88) | |  |
| Age, mean ± SD | 58.71 | 16.95 | 59.95 | 14.54 | 0.0787 |  | 63.52 | 13.79 | 63.39 | 13.57 | 0.0099 |
| Charlson Comorbidity Index, mean ± SD | 2.68 | 3.1 | 2.19 | 2.23 | 0.1797 |  | 3 | 3.21 | 2.52 | 2.31 | 0.1707 |
| LVEF, mean ± SD | 55.36 | 20.95 | 66.6 | 11.95 | 0.6589 |  | 60.57 | 16.89 | 61.77 | 14.64 | 0.0764 |
| CHA₂DS₂-VASc score, mean ± SD | 2.88 | 1.9 | 2.12 | 1.74 | 0.4191 |  | 2.91 | 2.13 | 2.65 | 1.94 | 0.1302 |
| Male | 20 | 58.82% | 4864 | 64.03% | 0.1072 |  | 14 | 60.87% | 53 | 60.23% | 0.0131 |
| Comorbidities |  |  |  |  |  |  |  |  |  |  |  |
| Hypertension | 17 | 50.00% | 3957 | 52.09% | 0.0419 |  | 13 | 56.52% | 48 | 54.55% | 0.0398 |
| Hyperlipidemia | 11 | 32.35% | 2359 | 31.06% | 0.0279 |  | 8 | 34.78% | 30 | 34.09% | 0.0146 |
| Diabetes mellitus | 6 | 17.65% | 1974 | 25.99% | 0.2030 |  | 6 | 26.09% | 18 | 20.45% | 0.1336 |
| Coronary artery disease | 12 | 35.29% | 2223 | 29.27% | 0.1292 |  | 8 | 34.78% | 34 | 38.64% | 0.0800 |
| Myocardial infarction | 2 | 5.88% | 530 | 6.98% | 0.0447 |  | 2 | 8.70% | 3 | 3.41% | 0.2231 |
| Congestive heart failure | 7 | 20.59% | 1151 | 15.15% | 0.1422 |  | 4 | 17.39% | 21 | 23.86% | 0.1605 |
| Atrial fibrillation | 3 | 8.82% | 586 | 7.71% | 0.0403 |  | 1 | 4.35% | 11 | 12.50% | 0.2967 |
| Venous thromboembolism | 6 | 17.65% | 60 | 0.79% | 0.6091 |  | 0 | 0.00% | 3 | 3.41% | 0.2657 |
| Cerebrovascular disease | 3 | 8.82% | 1270 | 16.72% | 0.2382 |  | 2 | 8.70% | 10 | 11.36% | 0.0889 |
| Peripheral artery disease | 0 | 0.00% | 85 | 1.12% | 0.1504 |  | 0 | 0.00% | 0 | 0.00% | 0.0000 |
| Chronic obstructive pulmonary disease | 3 | 8.82% | 1131 | 14.89% | 0.1885 |  | 3 | 13.04% | 11 | 12.50% | 0.0163 |
| Autoimmune disease | 1 | 2.94% | 239 | 3.15% | 0.0119 |  | 1 | 4.35% | 3 | 3.41% | 0.0486 |
| Chronic kidney disease | 5 | 14.71% | 1250 | 16.46% | 0.0483 |  | 4 | 17.39% | 16 | 18.18% | 0.0207 |
| Liver Cirrhosis | 0 | 0.00% | 93 | 1.22% | 0.1574 |  | 0 | 0.00% | 0 | 0.00% | 0.0000 |
| Cancer | 9 | 26.47% | 1090 | 14.35% | 0.3042 |  | 7 | 30.43% | 21 | 23.86% | 0.1482 |
| Medications |  |  |  |  |  |  |  |  |  |  |  |
| ACEi | 3 | 8.82% | 858 | 11.30% | 0.0822 |  | 2 | 8.70% | 4 | 4.55% | 0.1675 |
| ARB | 7 | 20.59% | 2010 | 26.46% | 0.1388 |  | 5 | 21.74% | 19 | 21.59% | 0.0036 |
| Direct oral anticoagulant | 1 | 2.94% | 143 | 1.88% | 0.0690 |  | 0 | 0.00% | 0 | 0.00% | 0.0000 |
| Warfarin | 5 | 14.71% | 189 | 2.49% | 0.4466 |  | 1 | 4.35% | 7 | 7.95% | 0.1505 |
| Antiplatelet | 10 | 29.41% | 2484 | 32.70% | 0.0711 |  | 8 | 34.78% | 27 | 30.68% | 0.0875 |
| Beta blocker | 6 | 17.65% | 1367 | 18.00% | 0.0091 |  | 3 | 13.04% | 17 | 19.32% | 0.1710 |
| Digoxin | 8 | 23.53% | 207 | 2.73% | 0.6475 |  | 3 | 13.04% | 16 | 18.18% | 0.1419 |
| Nondihydropyridine CCB | 2 | 5.88% | 524 | 6.90% | 0.0415 |  | 2 | 8.70% | 7 | 7.95% | 0.0268 |
| Statin | 5 | 14.71% | 1573 | 20.71% | 0.1577 |  | 5 | 21.74% | 15 | 17.05% | 0.1189 |
| Oral hypoglycemic agent | 6 | 17.65% | 966 | 12.72% | 0.1377 |  | 6 | 26.09% | 18 | 20.45% | 0.1336 |
| Follow-up (years), mean ± SD | 4.30 | 4.70 | 6.61 | 5.25 |  |  | 3.81 | 5.03 | 6.11 | 5.39 |  |

ACEi, angiotensin converting enzyme inhibitor; ARB, angiotensin receptor blocker; ASMD, absolute standardized mean difference; CCB, calcium channel blocker; SD standard deviation

**Supplemental Table 5.** Incidence rate of cardiovascular outcomes according to cardiac chamber of the thrombus

|  |  | Thrombus (+) | |  | Thrombus (-) | |  | Thrombus (+) vs. Thrombus (-) |
| --- | --- | --- | --- | --- | --- | --- | --- | --- |
|  | Outcome | Events | Incidence rate (95%CI) |  | Events | Incidence rate (95%CI) |  | Hazard ratio (95%CI); p value |
| LA thrombus | IS/SE | 88 | 4.81 (3.81 - 5.82) |  | 174 | 2.51 (2.13 - 2.88) |  | 1.85(1.44-2.36); p<0.0001 |
| vs. | Pulmonary embolism | 9 | 0.45 (0.2 - 0.85) |  | 22 | 0.3 (0.18 - 0.43) |  | 1.45(0.66-3.18); p=0.3572 |
| No thrombus | Myocardial infarction | 19 | 0.95 (0.52 - 1.38) |  | 52 | 0.72 (0.52 - 0.92) |  | 1.3(0.77-2.19); p=0.3288 |
|  | Heart failure | 151 | 8.81 (7.41 - 10.22) |  | 418 | 6.49 (5.87 - 7.12) |  | 1.3(1.09-1.56); p=0.0041 |
|  | Cardiac death | 86 | 4.24 (3.34 - 5.14) |  | 152 | 2.08 (1.75 - 2.41) |  | 2.02(1.55-2.64); p<0.0001 |
|  | MACE | 268 | 17.42 (15.34 - 19.51) |  | 628 | 10.24 (9.44 - 11.04) |  | 1.61(1.39-1.86); p<0.0001 |
|  |  |  |  |  |  |  |  |  |
| LV thrombus | IS/SE | 78 | 3.3 (2.57 - 4.04) |  | 189 | 2.36 (2.02 - 2.7) |  | 1.38(1.05-1.8); p=0.0201 |
| vs. | Pulmonary embolism | 6 | 0.24 (0.09 - 0.52) |  | 11 | 0.13 (0.05 - 0.21) |  | 1.79(0.66-4.82); p=0.2505 |
| No thrombus | Myocardial infarction | 89 | 3.84 (3.04 - 4.63) |  | 188 | 2.35 (2.02 - 2.69) |  | 1.58(1.23-2.04); p=0.0004 |
|  | Heart failure | 286 | 15.32 (13.54 - 17.09) |  | 708 | 10.44 (9.67 - 11.21) |  | 1.4(1.22-1.6); p<0.0001 |
|  | Cardiac death | 107 | 4.25 (3.45 - 5.06) |  | 267 | 3.19 (2.81 - 3.57) |  | 1.32(1.06-1.64); p=0.0147 |
|  | MACE | 386 | 22.29 (20.07 - 24.52) |  | 996 | 15.59 (14.62 - 16.56) |  | 1.36(1.21-1.53); p<0.0001 |
|  |  |  |  |  |  |  |  |  |
| RA thrombus | IS/SE | 3 | 1.13 (0.23 - 3.3) |  | 34 | 2.44 (1.62 - 3.26) |  | 0.43(0.13-1.42); p=0.1666 |
| vs. | Pulmonary embolism | 6 | 2.36 (0.86 - 5.13) |  | 6 | 0.41 (0.15 - 0.89) |  | 4.63(1.79-11.98); p=0.0016 |
| No thrombus | Myocardial infarction | 2 | 0.75 (0.09 - 2.72) |  | 10 | 0.68 (0.26 - 1.1) |  | 1.03(0.22-4.78); p=0.9714 |
|  | Heart failure | 21 | 9.06 (5.19 - 12.94) |  | 63 | 4.64 (3.5 - 5.79) |  | 1.7(1.01-2.85); p=0.044 |
|  | Cardiac death | 8 | 2.99 (1.29 - 5.89) |  | 21 | 1.42 (0.81 - 2.03) |  | 1.96(0.84-4.61); p=0.122 |
|  | MACE | 32 | 14.62 (9.55 - 19.68) |  | 99 | 7.72 (6.2 - 9.24) |  | 1.63(1.08-2.45); p=0.0198 |
|  |  |  |  |  |  |  |  |  |
| RV thrombus | IS/SE | 1 | 2.06 (0.05 - 11.46) |  | 6 | 2.14 (0.78 - 4.65) |  | 0.88(0.1-7.73); p=0.9089 |
| vs. | Pulmonary embolism | 1 | 2.03 (0.05 - 11.29) |  | 3 | 1.03 (0.21 - 3.01) |  | 1.88(0.19-18.62); p=0.5883 |
| No thrombus | Myocardial infarction | 0 | -- |  | 2 | 0.69 (0.08 - 2.49) |  | -- |
|  | Heart failure | 3 | 6.35 (1.31 - 18.56) |  | 11 | 4.06 (1.66 - 6.46) |  | 1.49(0.38-5.95); p=0.5682 |
|  | Cardiac death | 0 | -- |  | 3 | 1.03 (0.21 - 3) |  | -- |
|  | MACE | 4 | 8.47 (2.31 - 21.68) |  | 16 | 6.16 (3.14 - 9.18) |  | 1.31(0.38-4.49); p=0.6722 |

IS/SE, ischemic stroke/systemic embolism, MACE, major adverse cardiovascular event

Incidence rates are expressed per 100 person-years.

**Supplemental Table 6.** Incidence rate of cardiovascular outcomes according to left heart thrombus and right heart thrombus

|  |  | Thrombus (+) | | Thrombus (-) | | Thrombus (+) vs. Thrombus (-) |
| --- | --- | --- | --- | --- | --- | --- |
|  | Events | Events | Incidence rate (95%CI) | Events | Incidence rate (95%CI) | Hazard ratio (95%CI); p value |
| Left heart thrombus | IS/SE | 166 | 3.96 (3.36 - 4.57) | 363 | 2.43 (2.18 - 2.68) | 1.59(1.33-1.91); p<0.0001 |
| vs. | Pulmonary embolism | 15 | 0.33 (0.16 - 0.5) | 33 | 0.21 (0.14 - 0.28) | 1.54(0.83-2.85); p=0.1685 |
| No thrombus | Myocardial infarction | 108 | 2.5 (2.03 - 2.97) | 240 | 1.58 (1.38 - 1.78) | 1.54(1.23-1.93); p=0.0002 |
|  | Heart failure | 437 | 12.21 (11.06 - 13.35) | 1126 | 8.52 (8.02 - 9.02) | 1.37(1.23-1.53); p<0.0001 |
|  | Cardiac death | 193 | 4.25 (3.65 - 4.85) | 419 | 2.67 (2.42 - 2.93) | 1.57(1.33-1.86); p<0.0001 |
|  | MACE | 654 | 20 (18.47 - 21.53) | 1624 | 12.97 (12.34 - 13.6) | 1.46(1.33-1.6); p<0.0001 |
|  |  |  |  |  |  |  |
| Right heart thrombus | IS/SE | 4 | 1.27 (0.35 - 3.26) | 40 | 2.39 (1.65 - 3.13) | 0.5(0.18-1.4); p=0.1871 |
| vs. | Pulmonary embolism | 7 | 2.3 (0.93 - 4.74) | 9 | 0.51 (0.23 - 0.97) | 3.86(1.64-9.09); p=0.002 |
| No thrombus | Myocardial infarction | 2 | 0.64 (0.08 - 2.3) | 12 | 0.68 (0.3 - 1.07) | 0.88(0.2-3.95); p=0.8658 |
|  | Heart failure | 24 | 8.6 (5.16 - 12.05) | 74 | 4.55 (3.51 - 5.58) | 1.67(1.03-2.72); p=0.0377 |
|  | Cardiac death | 8 | 2.52 (1.09 - 4.97) | 24 | 1.36 (0.81 - 1.9) | 1.75(0.76-4.02); p=0.1901 |
|  | MACE | 36 | 13.53 (9.11 - 17.95) | 115 | 7.46 (6.09 - 8.82) | 1.59(1.07-2.35); p=0.0203 |

IS/SE, ischemic stroke/systemic embolism, MACE, major adverse cardiovascular event

**Supplemental Table 7.** Baseline characteristics of study patients with left atrial appendage (LAA) vs. without LAA

|  | Before matching | | | | |  | After matching | | | | |
| --- | --- | --- | --- | --- | --- | --- | --- | --- | --- | --- | --- |
|  | LAA (+) | | LAA (-) | | ASMD |  | LAA (+) | | LAA (-) | | ASMD |
|  | (n = 403) | | (n = 360) | |  |  | (n= 279) | | (n = 279) | |  |
| Age, mean ± SD | 63.42 | 14.15 | 66.45 | 13.25 | 0.2211 |  | 65.02 | 14.68 | 64.91 | 13.11 | 0.0082 |
| Charlson Comorbidity Index, mean ± SD | 2.63 | 2.16 | 2.6 | 2.17 | 0.0127 |  | 2.67 | 2.24 | 2.61 | 2.05 | 0.0300 |
| LVEF, mean ± SD | 58.84 | 15.11 | 59.26 | 14.48 | 0.0285 |  | 59.21 | 15.08 | 59 | 14.57 | 0.0142 |
| CHA₂DS₂-VASc score, mean ± SD | 3.17 | 1.97 | 3.23 | 1.92 | 0.0292 |  | 3.21 | 2.02 | 3.13 | 1.95 | 0.0416 |
| Male | 204 | 50.62% | 169 | 46.94% | 0.0736 |  | 137 | 49.10% | 137 | 49.10% | 0.0000 |
| Comorbidities |  |  |  |  |  |  |  |  |  |  |  |
| Hypertension | 207 | 51.36% | 178 | 49.44% | 0.0384 |  | 146 | 52.33% | 143 | 51.25% | 0.0215 |
| Hyperlipidemia | 141 | 34.99% | 106 | 29.44% | 0.1188 |  | 96 | 34.41% | 88 | 31.54% | 0.0610 |
| Diabetes mellitus | 98 | 24.32% | 78 | 21.67% | 0.0630 |  | 62 | 22.22% | 59 | 21.15% | 0.0261 |
| Coronary artery disease | 110 | 27.30% | 83 | 23.06% | 0.0978 |  | 73 | 26.16% | 72 | 25.81% | 0.0082 |
| Myocardial infarction | 15 | 3.72% | 17 | 4.72% | 0.0498 |  | 13 | 4.66% | 12 | 4.30% | 0.0173 |
| Congestive heart failure | 201 | 49.88% | 164 | 45.56% | 0.0866 |  | 139 | 49.82% | 131 | 46.95% | 0.0574 |
| Atrial fibrillation | 314 | 77.92% | 235 | 65.28% | 0.2830 |  | 205 | 73.48% | 201 | 72.04% | 0.0322 |
| Venous thromboembolism | 10 | 2.48% | 5 | 1.39% | 0.0794 |  | 7 | 2.51% | 5 | 1.79% | 0.0494 |
| Cerebrovascular disease | 159 | 39.45% | 131 | 36.39% | 0.0632 |  | 108 | 38.71% | 109 | 39.07% | 0.0074 |
| Peripheral artery disease | 9 | 2.23% | 7 | 1.94% | 0.0202 |  | 7 | 2.51% | 6 | 2.15% | 0.0238 |
| Chronic obstructive pulmonary disease | 75 | 18.61% | 73 | 20.28% | 0.0421 |  | 53 | 19.00% | 56 | 20.07% | 0.0271 |
| Autoimmune disease | 10 | 2.48% | 11 | 3.06% | 0.0350 |  | 10 | 3.58% | 9 | 3.23% | 0.0198 |
| Chronic kidney disease | 84 | 20.84% | 74 | 20.56% | 0.0071 |  | 61 | 21.86% | 59 | 21.15% | 0.0174 |
| Liver Cirrhosis | 4 | 0.99% | 4 | 1.11% | 0.0116 |  | 3 | 1.08% | 3 | 1.08% | 0.0000 |
| Cancer | 28 | 6.95% | 32 | 8.89% | 0.0719 |  | 24 | 8.60% | 22 | 7.89% | 0.0261 |
| Medications |  |  |  |  |  |  |  |  |  |  |  |
| ACEi | 56 | 13.90% | 65 | 18.06% | 0.1137 |  | 41 | 14.70% | 43 | 15.41% | 0.0200 |
| ARB | 134 | 33.25% | 116 | 32.22% | 0.0219 |  | 89 | 31.90% | 90 | 32.26% | 0.0077 |
| Direct oral anticoagulant | 77 | 19.11% | 35 | 9.72% | 0.2696 |  | 44 | 15.77% | 31 | 11.11% | 0.1369 |
| Warfarin | 186 | 46.15% | 154 | 42.78% | 0.0680 |  | 122 | 43.73% | 126 | 45.16% | 0.0289 |
| Antiplatelet | 191 | 47.39% | 129 | 35.83% | 0.2362 |  | 114 | 40.86% | 117 | 41.94% | 0.0218 |
| Beta blocker | 98 | 24.32% | 81 | 22.50% | 0.0429 |  | 63 | 22.58% | 62 | 22.22% | 0.0086 |
| Digoxin | 143 | 35.48% | 136 | 37.78% | 0.0476 |  | 99 | 35.48% | 98 | 35.13% | 0.0075 |
| Nondihydropyridine CCB | 81 | 20.10% | 76 | 21.11% | 0.0250 |  | 60 | 21.51% | 59 | 21.15% | 0.0088 |
| Statin | 93 | 23.08% | 60 | 16.67% | 0.1612 |  | 57 | 20.43% | 49 | 17.56% | 0.0731 |
| Oral hypoglycemic agent | 66 | 16.38% | 42 | 11.67% | 0.1360 |  | 38 | 13.62% | 32 | 11.47% | 0.0650 |
| Follow-up (years), mean ± SD | 6.04 | 4.90 | 5.72 | 5.49 |  |  | 5.79 | 4.96 | 6.00 | 5.53 |  |

ACEi, angiotensin converting enzyme inhibitor; ARB, angiotensin receptor blocker; ASMD, absolute standardized mean difference; CCB, calcium channel blocker; SD standard deviation

**Supplemental Table 8.** Incidence rate of cardiovascular outcomes according to left atrial appendage (LAA) thrombus (+) and LAA(-)

|  |  | LAA (+) | |  | LAA (-) | | LAA (+) vs. LAA (-) |
| --- | --- | --- | --- | --- | --- | --- | --- |
|  | Events | Events | Incidence rate (95%CI) |  | Events | Incidence rate (95%CI) | Hazard ratio (95%CI); p value |
| LAA (+) | IS/SE | 37 | 4.21 (2.85 - 5.56) |  | 40 | 4.82 (3.33 - 6.32) | 0.88(0.56-1.4); p=0.5953 |
| vs. | Pulmonary embolism | 3 | 0.31 (0.06 - 0.91) |  | 3 | 0.33 (0.07 - 0.96) | 0.96(0.19-4.86); p=0.9604 |
| LAA (-) | Myocardial infarction | 7 | 0.74 (0.3 - 1.52) |  | 8 | 0.87 (0.38 - 1.72) | 0.85(0.3-2.36); p=0.7507 |
|  | Heart failure | 75 | 9.2 (7.12 - 11.28) |  | 69 | 8.99 (6.87 - 11.11) | 1.04(0.74-1.45); p=0.8308 |
|  | Cardiac death | 35 | 3.63 (2.43 - 4.83) |  | 39 | 4.22 (2.89 - 5.54) | 0.87(0.56-1.35); p=0.5254 |
|  | MACE | 114 | 15.26 (12.46 - 18.07) |  | 123 | 18.01 (14.83 - 21.2) | 0.88(0.67-1.14); p=0.3246 |

IS/SE, ischemic stroke/systemic embolism, MACE, major adverse cardiovascular event
